# The State of Alaska's early experience with institutionalization of health impact assessment

**DOI:** 10.3402/ijch.v72i0.22101

**Published:** 2013-08-05

**Authors:** Paul J. Anderson, Sarah Yoder, Ed Fogels, Gary Krieger, Joseph McLaughlin

**Affiliations:** 1 Department of Health and Social Services, Division of Public Health, Section of Epidemiology, Anchorage, Alaska; 2 Alaska Department of Natural Resources, Anchorage, Alaska; 3 Newfields Associates, Boulder, Colorado

**Keywords:** health impact assessment, institutionalization, Alaska

## Abstract

**Background:**

Many nations routinely include health impact assessments (HIA) in public policy decisions. Institutionalization of HIA formally integrates health considerations into a governmental decision-making process. We describe an example of institutionalization in the United States through Alaska's early experience with institutionalization of HIA.

**Literature review:**

HIA arose from a series of health conferences in the 1970s that affirmed the importance of “health for all.” A number of key milestones eventually defined HIA as a unique field of impact assessment. There are several approaches to institutionalization, and one common approach in the United States is through the National Environmental Policy Act (NEPA). NEPA formed the basis for the earliest HIAs in Alaska.

**Program description:**

Early HIAs in Alaska led to conferences, working groups, a state guidance document and the institutionalization of a HIA program within the Department of Health and Social Services in 2010. A medical epidemiologist staffs the program, which utilizes contractors to meet rising demand for HIA. The HIA program has sustainable funding from the state budget and from the state's natural resource permitting process. The HIA document is the main deliverable, but the program performs other tasks, including fieldwork and technical reviews. The HIA program works closely with a host of collaborative partners.

**Conclusion:**

Alaska's institutionalized HIA program benefits from sustainable funding that promotes continuous quality improvement and involves the program in the entire life cycle of a development project. The program structure adapts well to variations in workflow and supports a host of quality control activities. Currently, the program focuses on HIAs for natural resource development projects.

Health impact assessment (HIA) is a “combination of procedures, methods and tools by which a policy, program, or project may be judged as to its potential effects on the health of a population, and the distribution of those effects within the population” (1). Institutionalization of HIA means that HIA has become a systematic part of the decision-making process (2). The institutionalization of HIA is advanced in Australia, Canada and the European Union, where many governments require HIA for natural resource development projects, urban planning projects or public policy decisions.

Comparatively, the institutionalization of HIA is slower in the United States. A variety of groups have completed HIAs for urban planning actions, policy formation and the permitting of large-scale natural resource development projects (3). Most HIAs in the United States are grant-funded, but some federal agencies have made HIA a “best practice” and a few states have taken steps towards HIA legislation (4). Institutionalization of HIA remains a topic of national discussion and an option considered by some state and local governments. Where state and federal agencies do maintain HIA capacity, the work is primarily grant-funded and/or project-specific.

Several key institutions such as the US Centers for Disease Control and Prevention (CDC), the Health Impact Project (Robert Wood Johnson Foundation and The Pew Charitable Trusts), and the University of California, Los Angeles (UCLA) continue to stimulate tremendous growth in HIA capacity throughout the United States.

## HIA and institutionalization

Reiner Banken located the origins of HIA in a declaration of “health for all” at the International Conference on Primary Health Care in Almaty, Kazakhstan, in 1978 (formerly Alma-Ata, Kazakh Soviet Socialist Republic). Since that time, a series of milestones have been recognized as markers for the development of HIA as a unique field of study and work (Table I).

**Table I:** Some early milestones in HIA (1981–2000) (5)

1981	Establishment of PEEM (Panel of Experts in Environmental Management for vector control) by WHO, FAO and UNEP.
1984	Start of the HIA component as part of annual EIA training at the Centre for Environmental Impact Assessment and Management in Aberdeen (partly sponsored by WHO Europe). Annual sessions continued up to the beginning of the 1990s.
1986	WHO meeting on the Health and Safety component of environmental impact assessment (6).
1988	Analysis of the methodological and substantive issues affecting human health considerations by the Monitoring and Assessment Research Centre, London (7).
1989	First edition of the Guidelines for forecasting the vector-borne disease implications of water resources development by PEEM (8).
1991	Survey on HIA/EIA practice in Canada (9).
1992	Handbook for practitioners on environmental and health impact assessment of development projects (10).
	Asian Development Bank guidelines for the health impact assessment of development projects (11).
1993	Quebec framework for HIA/EIA, including a section on social impact assessment (12).
1994	Australian national framework for environment and health impact assessment (13).
	Publication of the German framework on HIA/EIA (14).
1997	Update on HIA in the Environmental Assessment Sourcebook of the World Bank (15).
1998	Publication on health and environmental impact assessment by the British Medical Association (16).
	HIA section at the International Association for Impact Assessment.
1999	Gothenburg Consensus Paper on HIA.
2000	The Canadian handbook on health impact assessment – a work in progress (17).
2000	Memorandum of understanding between the International Association for Impact Assessment and the World Health Organization.

Since 2000, HIA practice has rapidly expanded and produced several international standards, national guidance documents and the eventual institutionalization of HIA in some countries (17).

Institutionalization of HIA may be accomplished by a variety of means that include: 1) proposing HIA as a useful, easy and powerful tool to decision-makers 2) implementing administrative frameworks that bind different institutions and 3) creating legal frameworks that function as permanent rules (5).

The US National Environmental Policy Act 1969 (NEPA) requires that federal agencies prepare a statement of environmental impacts (EIA/EIS) whenever a proposed development project could significantly affect the human environment in an area under their jurisdiction. NEPA is an example of how an impact assessment process can become rapidly institutionalized through legal frameworks. While many federal agencies may have some jurisdiction for a given project, 1 federal agency serves as the lead that oversees the EIS process and has the final record of decision. The consideration of human health impacts is a required component of the EIS, but this component has been largely discounted in the past, prompting the need for a more dedicated and comprehensive process to address human health – the HIA.

## Institutionalization of HIA in the United States

In the United States, some of the initial HIA literature describes how HIA might be institutionalized within NEPA, particularly in states like Alaska that contain large reserves of natural resource wealth. Several authors have traced the history of the NEPA process in the United States and concluded that human health was not carefully considered in early EIA/EIS documents, even though the documents were considered legally acceptable (18,19). Environmental topics related to wildlife, water quality and air quality received lengthy treatment while public health was often limited to a small section of the overall document. By 2007, Wernham had described the first HIA integrated into the NEPA process for proposed oil and gas development in Alaska's North Slope region (20). Numerous US states, including Alaska, have pursued a range of approaches to institutionalization, including administrative and more compulsory legal frameworks (21).

## Institutionalization of HIA in Alaska's State Government

In 2008, early work in Alaska culminated with a HIA conference that gathered experts from federal agencies, CDC, state and local government, regional health corporations, the Alaska Native Tribal Health Consortium (ANTHC) and respected national and international HIA practitioners. Conference attendees established a working group that developed a HIA toolkit to provide technical guidance for HIA practice in Alaska (available at: www.epi.alaska.gov/hia). Working group participants also identified the need for 1 agency to maintain and update the HIA toolkit, respond to public feedback and develop Alaska's capacity for HIA. In response to this need and with support from partners, the Alaska Department of Health and Social Services (DHSS) established a HIA program in July 2010. The HIA program exists within the Alaska Section of Epidemiology. HIA is not legally required in Alaska, but it is increasingly viewed as a “best-practice” approach to natural resource development in the state.

### Purpose

The purpose of the Alaska HIA program is to ensure that large-scale resource development projects are designed to maximize the positive health benefits and to minimize the negative health impacts to all Alaskans.

### Staff

A full-time medical epidemiologist serves as the HIA program manager and is supported by 1 part-time administrative member of staff and a master's degree-level epidemiologist supported by CDC and the Council of State and Territorial Epidemiologists (CSTE).

### State guidance document

The HIA program maintains the *HIA Toolkit* (www.epi.alaska.gov), which describes the history of HIA in the state and provides guidance for topics such as stakeholder engagement, best data practices, subsistence research and the correct use of health data. HIA practitioners can refer the toolkit as they prepare documents, plan fieldwork and provide editorial comments. The HIA program invites stakeholder comments on the HIA toolkit and will update the document periodically to reflect the most current practice standards.

### Funding

HIA work is funded through financial agreements made during the NEPA process. Under NEPA, a designated federal agency is responsible for preparing an Environmental Impact Statement (EIS) for a proposed development project on federal lands. In Alaska, this is the most common context for performing a HIA. Applicants often coordinate their state permits through a financial agreement with the Office of Project Management and Permitting (OPMP) in the Alaska Department of Natural Resources (DNR). If the HIA program is asked to perform a HIA on a NEPA project, the HIA is funded through a reimbursable service agreement with DNR's OPMP. While this is the primary funding source for the HIA program, some programmatic work is also partly supported by state general funds.

### Workflow

The HIA program manages the large workflow needs through the support of a HIA contractor. This arrangement is highly scalable, removes the need for expansion of government, and provides the program with a deep pool of HIA expertise. HIA contractors gather data, execute fieldwork, review literature, prepare documents and respond to comments as a part of the NEPA process. The HIA program manager oversees this work and facilitates relationships between the contractors and the many stakeholders for the project. The HIA program also reviews work products from its own contractors and other contractors who perform HIA in Alaska.

### Work products

Currently, the HIA program produces a “standalone HIA,” which becomes an appendix to the larger EIS document. An EIS contractor references the standalone HIA to write the public health sections of the EIS. This allows the HIA to organize all data, public comments, impacts and recommendations by widely accepted health effect categories (HECs) that systematically address a set of health outcomes and determinants. A standalone document consolidates all data, comments, analyses and conclusions into one comprehensive document. A standalone HIA is not a requirement of the program, but rather is one approach to simplify the research and writing of an EIS that adequately addresses human health.

The HIA program does not take wholesale positions “for or against” projects; rather, it provides decision-makers with a forecast of potential health impacts that are most likely to occur if the proposed project were permitted. The HIA does explore mitigation strategies, but the NEPA/EIS process ultimately determines mitigations.

### Partnerships

The HIA program collaborates closely with a wide network of stakeholders, including federal, state, local and tribal agencies, as well as project proponents and community councils.

Regional Alaska Native health entities such as the Yukon-Kuskokwim Health Corporation and the North Slope Borough DHSS join with the HIA Program to review HIA documents, coordinate field work and educate the public regarding HIAs being performed in their respective communities. The HIA program also relies heavily on health data from the ANTHC and maintains close working relationships with ANTHC staff and researchers.

Collaborations with applicants start during baseline data collection through meetings that convey important HIA findings and updates. Applicants assist the HIA team with field visits to their facilities and share valuable information gained from their community engagement work. While applicants exchange information with the HIA program, they do not edit work products from the HIA program during this period unless the public also reviews the document.

Collaboration with federal agencies and the cooperating agencies in the NEPA process (typically the state agencies, tribal governments and local governments) occurs through weekly project updates and community scoping meetings that are part of the NEPA process. Cooperating agencies also travel with the HIA program for some community visits to integrate the health team into the NEPA process.

Collaboration with state agencies such as the departments of Environmental Conservation, Fish and Game, Natural Resources and Transportation also occurs though a wide variety of venues. For example, the HIA program manager participates in the weekly OPMP meetings, and functions as part of the DNR’ large mine permitting team (LMPT), and as a departmental liaison for the State Pipeline Coordinators Office (SPCO). Work with the other state agencies is typically more project-specific.

## Conclusion

In Alaska, HIA work is a systematic part of the impact analysis and permitting process for large-scale natural resource development projects. One major advantage of the program is that it has sustainable funding, primarily through the state's permitting process. Sustainable funding means that the HIA program can be involved from early project exploration to the project operation period. Consistent financial support also allows for continuous quality improvement in HIA program policies, the HIA toolkit, data gathering methods and stakeholder communication practices. Sustainable funding also allows simultaneous involvement with a variety of projects that could combine in ways that intensify expected health benefits or negative health consequences for Alaskans.

Another advantage of the program is its flexibility. The HIA program currently oversees assessment work on numerous large-scale natural resource development projects at various stages of exploration and permitting. The demand for HIA project management, fieldwork and technical services varies greatly depending on the project stage. The use of contractors provides a highly skilled resource that creates a small governmental footprint and provides flexible capacity to meet varying demands.

Quality assurance is another advantage of the HIA program. The program assures HIA quality through a formal guidance document (the HIA toolkit), internal review, subject matter expert reviews, and public comment/response cycles. The HIA program also assures quality through technical review of NEPA documents under consideration by the LMPT or the SPCO.

Current HIA work in Alaska is limited to large natural resource development projects. The program has yet to work on HIAs related to public policy changes or urban planning proposals. The small size of the HIA program also limits its range of services and limits the program's ability to support a number of HIA educational and research activities needed in the state.

The institutionalization process in Alaska has produced several lessons that may benefit others interested in formalizing the use of HIA.

1. The NEPA process provides an excellent context for HIA because it prioritizes transparency, streamlines community engagement, integrates field research efforts and supplies key technical information for the HIA.
2. A HIA Toolkit can provide useful guidance for practitioners, a transparent basis for quality assurance and an avenue for stakeholder input to improve the HIA process.
3. Institutionalization has produced interdependent activity between tribal, state and federal entities, and also advances efforts to promote public health.
4. Institutionalization has promoted public awareness of health issues and has also increased health knowledge among decision-makers.

In summary, HIA work has become a systematic part of the impact analysis and permitting process in Alaska for large-scale natural resource development projects. The State of Alaska's model might be useful to other governments who wish to institutionalize HIAs within their respective agencies.
